# Supplementary material for: Learning curve and surgical outcome of robotic assisted colorectal surgery with ERAS program
Source: Sci Rep. 2022 Nov 29;12:20566. doi: 10.1038/s41598-022-24665-w (PMC9709162; doi:10.1038/s41598-022-24665-w)
Supplement: Supplementary file 1 — Supplementary Information 1. [file 41598_2022_24665_MOESM1_ESM.zip › LSC Raw data-Ñ[▒K/128/P_ñw╝╨░O▒Kñσ.pdf]

[redacted]

Pathologic diagnosis:
Colon, terminal ileum, and appendix, total colectomy --- Staus post-polypectomy. Multiple tubular adenomas and tubulovillous adenomas with focal high grade dysplasia, involving the whole colorectum. The appendix, ileocecal valve, bilateral cut ends and mesocolic lymph nodes (0/77) are free of malignancy.

Ancillary study for diagnosis:
Deep cut done on section F.

Prognostic and predictive factor:
Combined S1018243
1. Tumor size: 0.8 cm.
2. Depth of invasion: Submucosa.
3. Histologic grade: G2: Moderately differentiated.
4. Angiolymphatic invasion: Not identified.
5. Perineural invasion: Not identified.
6. Tumor deposits (discontinuous extramural extension): Not identified.
7. Macroscopic tumor perforation: Not identified.
8. Surgical Margins: a) Proximal Margin: Uninvolved by invasive carcinoma. b) Distal Margin: Uninvolved by invasive carcinoma. c) Radial or Mesenteric Margin: Uninvolved by invasive carcinoma.
9. Lymph node status: Uninvolved (0/77).
10. Extranodal involvement: Not identified.
11. Tumor regression grading S/P CCRT: No known presurgical therapy.
12. Type of polyp in which invasive carcinoma arose: Tubulovillous adenoma.
13. Additional pathologic findings: Adenoma(s).
14. Pathological TNM Stage: pT1N0(According to the eighth edition, American Joint Committee on Cancer Staging Guidelines for Tumors).
15. TNM descriptors: Not applicable.

Gross description:
The specimen consists of a totally resected colon with anus, 73 cm in length and 7 cm in distal circumference, terminal ileum, 11 cm in length and 5 cm in circumference, an unremarkable appendix, 5x1x1 cm in length, omentum, 25x10x1 cm, and mesocolic and perirectal soft tissue. Three are two pedunculated polypoid tumors and one gray tan firm flat tumor, 2x1.5x1 cm (tumor 1) and 1x0.5x0.5 cm (tumor 2) and 1.5x1x0.5 cm (tumor 3), are noted, 10 cm, 41 cm and 55 cm in distance to the distal cut end, respectively. There are also numerous polyps over the whole colorectal mucosa. The both cut ends are free of tumor or polyps grossly. Representative part for section: A) colon cut end B) small intestine cut end C) ileocecal valve D) appendix E-F) tumor 1 G) tumor 2 H-J) tumor 3 K-T) numerous polyps over colon U) small intestine V-AB) lymph nodes.
#T-59420_2 #M-82110_2 0 1105 000000
#T-C4560_2 #M-09183_2 0 1105 000000
#T-59200_2 #M-09183_2 0 1105 000000

已電子簽章

敬告：本單僅供醫師參考使用，不具任何申請效力！！
請病患妥為保管，若不慎遺失需自負個人資料外洩責任！
